# Closing two doors of viral entry: Intramolecular combination of a coreceptor- and fusion inhibitor of HIV-1

**DOI:** 10.1186/1743-422X-5-56

**Published:** 2008-05-01

**Authors:** Erhard Kopetzki, Andreas Jekle, Changhua Ji, Eileen Rao, Jun Zhang, Stephan Fischer, Nick Cammack, Surya Sankuratri, Gabrielle Heilek

**Affiliations:** 1 Pharmaceuticals Division, Roche Penzberg, Penzberg, Germany; 2 Virology Diseases Area, Roche Palo Alto, 3431 Hillview Ave., Palo Alto, CA, USA

## Abstract

We describe a novel strategy in which two inhibitors of HIV viral entry were incorporated into a single molecule. This bifunctional fusion inhibitor consists of an antibody blocking the binding of HIV to its co-receptor CCR5, and a covalently linked peptide which blocks envelope mediated virus-cell fusion. This novel bifunctional molecule is highly active on CCR5- and X4-tropic viruses in a single cycle assay and a reporter cell line with IC_50 _values of 0.03–0.05 nM. We demonstrated that both inhibitors contribute to the antiviral activity. In the natural host peripheral blood mononuclear cells (PBMC) the inhibition of CXCR4-tropic viruses is dependant on the co-expression of CCR5 and CXCR4 receptors. This bifunctional inhibitor may offer potential for improved pharmacokinetic parameters for a fusion inhibitor in humans and the combination of two active antiviral agents in one molecule may provide better durability in controlling the emergence of resistant viruses.

## Introduction

Enveloped viruses, such as HIV-1, utilize membrane bound fusion proteins to mediate attachment and entry into specific target host cells. The viral entry process for HIV-1 has been well studied [1-3] and can be briefly described as the following sequence of steps: The initial contact between the virus and the host cell is established with the binding of the viral envelope glycoprotein (gp) gp120 to the cellular receptor CD4, this allows for the second binding step between gp120 and a co-receptor, CCR5 or CXCR4, respectively. The binding to the co-receptor triggers a conformational change of the viral envelope proteins and allows for the smaller envelope subunit gp41 to be inserted into the host membrane. This is followed by condensation of two helical regions within gp41, resulting in formation of a six helix bundle, facilitating close contact of the viral and host membranes and followed by fusion of the viral envelope with the cell membrane.

The choice of the co-receptor involved in the fusion process has given rise to the definition of viral tropism. Viruses using CCR5 are defined as R5 tropic, viruses using CXCR4 as X4-tropic and viruses being able to use both as dual or mixed tropic [4].

It has been well established that R5-tropic viruses are nearly exclusively present during the acute infection with HIV-1 and the asymptomatic phase, whereas X4-tropic viruses emerge in later phases of HIV infection and are associated with a more dramatic CD4 cell decline and progression towards AIDS [5,6].

Naturally occurring anti-CCR5 antibodies have been found in sero-negative partner of HIV-seropositive individuals [7] and in long-term non-progressors [8], suggesting that they may participate both in protection and in the control of HIV infection [9]. In fact this observation, and perhaps not the protection of antibodies in non-progressors led various companies to be interested in developing CCR5 antibodies.

Several companies have reported CCR5 monoclonal antibodies with pre-clinical and/or clinical proof-of-concept studies. Clinical proof of antiviral activity has been demonstrated for PRO-140 developed by Progenics Pharmaceuticals [10,11] and CCR5 mAb004 from Human Genome Sciences [12,13]. The Roche CCR5 antibody and its pre-clinical characterization have been described previously [14].

Due to the multi-step nature of the HIV entry, one can rationalize that combining a coreceptor inhibitor, such as a CCR5 antibody, with a fusion peptide, such as enfuvirtide (ENF), into one molecule might be an advantageous approach to prevent entry of HIV to the host cells at multiple steps. Scientific proof of such a synergistic mechanism has been demonstrated *in vitro *by drug-drug combination studies with CCR5 antibodies and ENF [15,16].

Here we describe a series of experiments using a novel HIV entry inhibitor, consisting of a CCR5 antibody that has been covalently linked to a fusion peptide inhibitor. The approach is aimed primarly to enhance the pharmacokinetic properties of the fusion peptide by covalent linkage to an antibody. In addition, this approach allowed us to explore the potential synergy of inhibition of HIV entry.

## Results

### Antiviral activity of the bifunctional HIV-entry inhibitor

The short plasma half-life of ENF requires twice daily injections [17], this dosing inconvenience has markedly limited the broader use of ENF. In an attempt to improve the *in vivo *pharmacokinetic properties a prototypic recombinant antibody-FI fusion protein was generated, in which two T-2635 fusion inhibitors were covalently linked to the C-terminal ends of the two heavy chains of a monoclonal antibody against the insulin-like growth factor-I receptor (IGF-IR). IGF-IR is a cell surface protein that is not involved in the HIV entry process. T-2635 is a helix-stabilized second generation FI with antiviral activity against virus strains resistant to ENF [18]. The antiviral potency of this construct (IGF-IRmAb-FI) was determined in a single cycle entry assay using virus particles generated by pseudotyping the labstrain NL4-3 (Δenv) with the envelope of the CCR5-tropic virus NL-Bal. Although IGF-IRmAb-FI showed antiviral activity, it was about 160-fold less active than T-2635 on a molar basis. As expected, the parental IGF-IR mAb had no activity up to 100 nM tested (Table 1). Several variants of IGF-IRmAb-FI with altered linkers and/or positions of fusion peptide attachment, heavy or light chain antibody components were also explored and none of them yielded substantial improvement in antiviral activity (data not shown).

**Table 1:** Antiviral activities of HIV inhibitors*

Ab/fusion inhibitors	IC_50 _± SD (nM)	
	NL-Bal (R5)	NL4-3 (X4)
T-2635	2.6 ± 0.6	19.1 ± 7.3
IGF-1RmAb	> 100	>100
IGF-1RmAb-FI**	421 ± 148	Not tested
CCR5mAb	0.9 ± 0.6	>100
BFFI (CCR5mAb-FI)**	0.03 ± 0.02	0.05 ± 0.0002

* Results are from two or more independent experiments.

** Two T-2635 fusion inhibitors fused to the C-terminal ends of the heavy chain of an anti-IGF-1R mAb or anti-CCR5mAb through {G(Q)_4_}_3_-NN linker.

**Figure 1:**
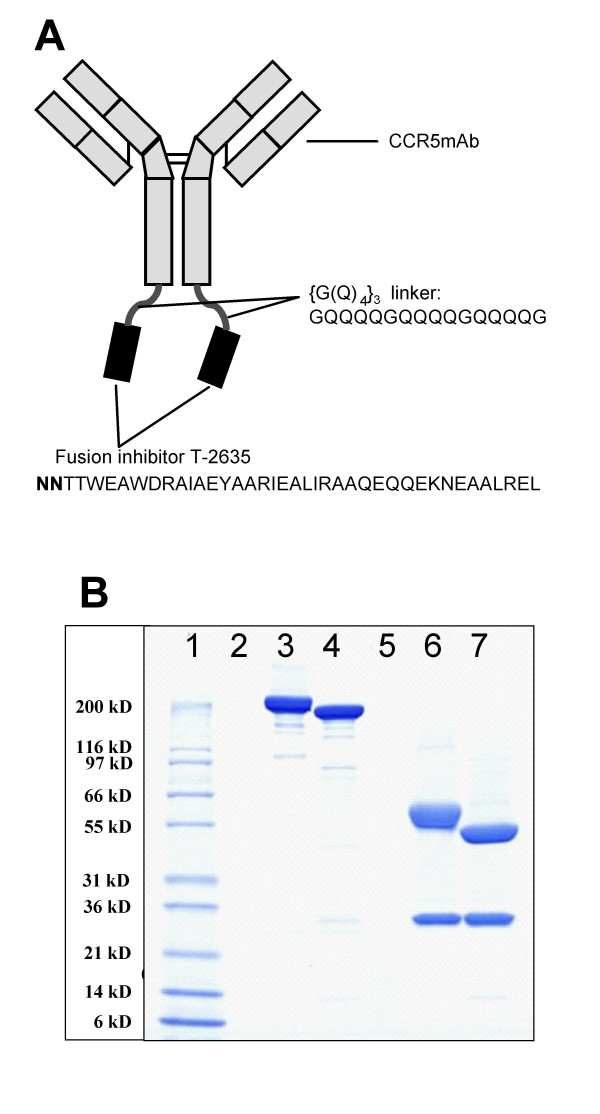
Design and biochemical characterization of BFFI. **A**. Schematic diagram of BFFI. BFFI is composed of the CCR5mAb RO-Ab0630, with two covalently attached T-2635 fusion inhibitors via {G(Q)_4_}_3 _linkers. The peptide sequences for the linker and T-2635 are shown. **B**. SDS-PAGE of BFFI and CCR5mAb. The lanes contain the following samples: Sample Buffer (lane 2, 5); MW Marker (lane 1); BFFI (lane 3); CCR5mAb (lane 4); reduced BFFI (lane 6); reduced CCR5mAb (lane 7).

We have previously described *in vitro *drug-drug combination studies between a CCR5mAb and ENF resulting in a synergistic antiviral effect against HIV-1 [16]. We hypothesized that a CCR5mAb-FI fusion protein may possess intramolecular synergy due to two components of the entry process involved and thus may potentially be more potent then the constructs containing the IGF-IRmAb. A chimeric human/mouse CCR5mAb that contains the variable regions of the highly potent mouse anti-human CCR5mAb ROAb14 and the human IgG1 scaffold was used to generate the chimeric mAb-FI protein. This bifunctional fusion inhibitor (BFFI) was expressed transiently in human embryonic kidney cells and purified to greater than 95% purity as demonstrated by SDS-PAGE (Fig. 1B) and analytical size-exclusion chromatography (data not shown). SDS-PAGE also verified the correct composition and size of the BFFI (Fig. 1B). BFFI was more potent at inhibiting HIV-1 in the single cycle antiviral assay against the pseudotyped R5 virus NL-Bal than either the fusion inhibitor T-2635 or the CCR5mAb alone. As shown in Table 1, BFFI is approximately 86-fold more potent than T-2635 and 30-fold more potent than CCR5mAb. Similar results were obtained when BFFI was tested against particles pseudotyped with the envelope of the X4 virus NL4-3 (Table 1).

### Mechanism of action of BFFI

**Figure 2:**
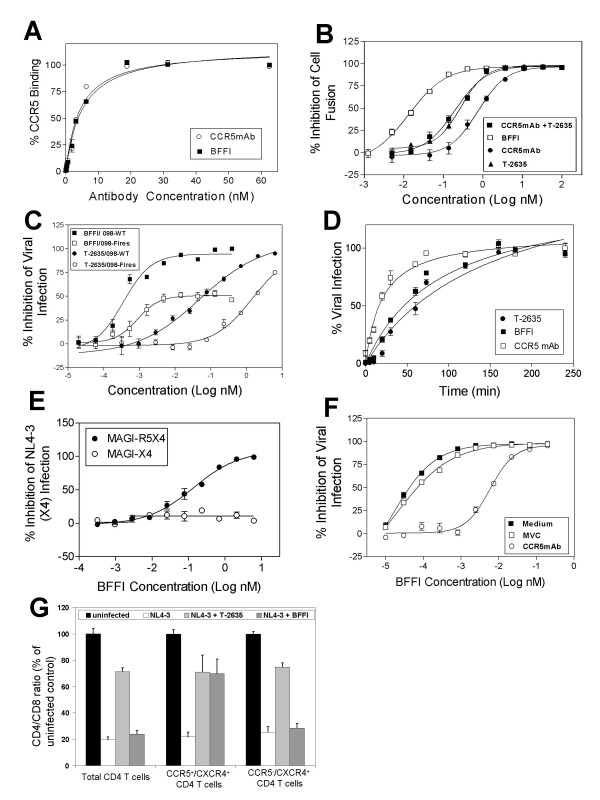
Mechanism of action for BFFI. **A**. BFFI (■) and CCR5mAb (○) bind to CCR5 with similar affinity as determined by FACS analysis. **B**. Antiviral Potency of BFFI. BFFI (□) has a higher antiviral potency than a 1:2 mixture of CCR5mAb with T-2635 (■) or either T-2635 (▲) or CCR5mAb (●) alone. Antiviral potency was determined in a single cycle entry assay using virus particles pseudotyped with the envelope of the CCR5-tropic virus NL-Bal. **C**. Reduced antiviral activity of BFFI against a partially T-2635-resistant virus. The antiviral activity of BFFI (squares) and T-2635 (circles) against wt (filled symbols) and the partially T-2635-resistant virus 098-FI^res ^(open symbols) was determined in an antiviral assay using replication-competent virus. **D**. Synchronized viral infection experiment. Cells were infected at 4°C, washed and warmed to 37°C. T-2635 (●), CCR5mAb (□) or the BFFI (■) were added at indicated time points. **E**. Antiviral activity of BFFI against X4 viruses is dependent on CCR5 expression. Antiviral activity of BFFI against virus particles pseudotyped with the envelope of the X4-tropic virus NL4-3 was determined using MAGI cells expressing CXCR4 (○) or CXCR4 and CCR5 (●). **F**. Antiviral activity of BFFI against X4 virus particles is dependent on CCR5 binding. Cells were pre-incubated for 45 minutes with the CCR5 antagonist maraviroc (MVC, □), the CCR5mAb (○) or medium (■), washed and then infected with virus particles pseudotyped with the envelope of the X4-tropic virus NL4-3 in presence of BFFI. **G**. BFFI protects CCR5-expressing CD4(+) T-cells from NL4-3 virus cytopathic effect. PBMC were infected with NL4-3 virus in presence of either T-2636 or BFFI. After 5 days of incubation, depletion of total CD4(+) T-cells, CCR5^+ ^CD4 T-cells and CCR5^- ^CD4 T-cells was measured by flow cytometry and displayed as the ratio of CD4 to CD8 T-cells.

In order to understand the mechanism for the antiviral potency of BFFI and the contribution of the individual inhibitors to viral entry, we performed a series of experiments. To test the possibility of an increased affinity of BFFI to the CCR5 receptor as the source of the increase in antiviral activity, saturation binding experiments were performed. BFFI and CCR5mAb had very similar binding affinity to human CCR5, suggesting that addition of the FI did not alter the binding affinity of the CCR5 antibody component (Fig. 2A). We tested mixing of CCR5mAb and T-2635 at a 1:2 ratio and observed only marginal additive antiviral activity with an approximately 2-fold difference in IC_50 _(Fig. 2B). These data suggest that the antiviral potency of BFFI cannot be explained by increased binding to CCR5 or to additive effects between two independent pharmacophores, but may result from intramolecular synergy.

We designed several experiments to further explore if both pharmacophores in BFFI are indeed effective in blocking viral entry. Comparing the antiviral activity of BFFI to IGF-IRmAb-FI, BFFI was 15,000-fold more active than IGF-IRmAb-FI (Table 1). Since the only difference between the two fusion proteins is the variable region of the antibody, CCR5 recognizing versus IGF-1 receptor recognizing, these result suggests that the variable region of the CCR5mAb in BFFI contributes to the antiviral activity.

Considering the contribution of the FI, the antiviral activity of BFFI against X4 virus in the single cycle antiviral assay (Table 1) suggest that the FI T-2635 was active in blocking viral entry as the CCR5 mAb alone has no activity against X4 viruses. To further confirm the antiviral activity of the FI portion, BFFI was tested against T-2635-resistant, replication competent, X4 virus 098-FI^res^. In viral infectivity assays, 098-FI^res ^showed a 25-fold reduction of susceptibility to T-2635 in comparison with the parent isolate 098 (Fig. 2C). We hypothesized that if the FI portion of BFFI contributed to the overall antiviral potency, the reduced antiviral activity of T-2635 against 098-FI^res ^should be reflected in a similar susceptibility loss against BFFI. Indeed, the 098-FI^res ^susceptibility to BFFI was reduced approximately 32-fold compared to 098 (Fig. 2C). In addition, maximal percent inhibition (MPI) of 098-FI^res ^infection by BFFI was found to be 52% (Fig. 2b), similar to previously described findings when loss of susceptibility to other entry inhibitors has been reported [19,20]. HIV isolates 098 and 098-FI^res ^are X4-tropic and, therefore, viral fusion can not be suppressed by the CCR5mAb fragment within BFFI. These results suggest that T-2635 FI of BFFI contributed to the antiviral activity of BFFI.

Studies of the sequential steps of viral envelope fusion with the host cell established that CCR5 inhibitors block HIV entry at an early stage, while HIV FIs work at a later stage [16,21]. In vitro these processes can be studied in synchronized viral infection experiments where viral particles are allowed to absorb to the cell surface but later steps are arrested by low temperature treatment [22]. We were interested to apply this technique to determine time to effect for the CCR5 mAb, BFFI and T-2635. To test the timing of HIV entry inhibition, MAGI-R5 cell infection with R5-tropic pseudotyped virus was synchronized by spinoculation at 4°C, which allows binding of virus to the cells, but blocks subsequent steps. Synchronized HIV infection was triggered by adding medium and/or inhibitors and warming to 37°C. This technique allows to determine the latest possible time of action of different classes of entry inhibitors. As expected, CCR5mAb maximally inhibited HIV entry at an early stage (t_1/2 _= 18 min) and T-2635 inhibited HIV entry at a late stage (t_1/2 _= 48 min). BFFI inhibited HIV entry at a time point of t_1/2 _= 40 min (Fig. 2D) which would suggest that the FI components in BFFI are the major contributors for the rate determining inhibition.

### Antiviral potency of BFFI against X4-tropic viruses is dependent on CCR5 co-expression on target cells

We showed that BFFI is active against R5- and X4-tropic viruses in a single cycle entry assay, which utilizes a cell line expressing high levels of CCR5, CXCR4 and CD4 receptors. To expand the utility of the BFFI molecule beyond a scientific concept to a more clinically relevant setting, we wanted to confirm these results in the natural target cells for HIV, human peripheral blood mononuclear cells (PBMC). With five replication competent CCR5-tropic viral isolates tested, BFFI showed similar potency to the CCR5mAb and T-2635 (Table 2). There was a trend in the mean value for these 5 viruses for BFFI to show approximately 2 fold improved potency compared to the individual components, but more viral isolates and possibly infection macrophages would need to be tested to confirm these findings.

**Table 2:** Antiviral activity IC_50 _of BFFI in PBMC assays

HIV Virus	Tropism	BFFI*	CCR5mAb*	T-2635*
NLBal	R5	0.13 ± 0.06	0.23 ± 0.09	0.58 ± 0.40
JRCSF	R5	0.60 ± 0.13	1.16 ± 0.45	0.29 ± 0.10
YU2.c	R5	0.07 ± 0.016	0.24 ± 0.09	0.26 ± 0.12
92US715	R5	0.34 ± 0.19	0.96 ± 0.39	0.55 ± 0.30
CC1/85	R5	0.67 ± 0.36	1.12 ± 0.34	1.41 ± 0.31
**Mean**	**R5**	**0.36 ± 0.27**	**0.62 ± 0.47**	**0.74 ± 0.47**
p-value vs. BFFI			0.0800	0.0410
NL4-3	X4	>100	>100	0.70 ± 0.31
89.6	R5X4	>100	>100	0.91 ± 0.12

* Results are IC_50 _± SD (nM) from 3 or more independent experiments.

The log10 values of the IC_50 _for all three drugs and R5 virus isolates were compared using a two-way ANOVA model including terms of drug and virus followed by post-hoc comparison using Fisher's LSD test

While BFFI is active against X4-tropic viral particles in the single cycle entry assay, no antiviral activity was detected for the X4-tropic (NL4-3) or dual tropic (89.6) virus up to 100 nM tested, in the PBMC assay (Table 2).

Human PBMC are a mixture of cells with variant expression of cell surface receptors. Only a small subset of CD4(+) T-cells express CCR5, while most CD4(+) T-cells express CXCR4 [23,24]. We hypothesized that the activity of BFFI against X4-tropic viruses is dependent on the co-expression of CCR5 on the target cells. This model would explain why BFFI is active against X4 tropic virus in cell lines with high CCR5 expression, while being inactive in PBMC with their limited CCR5 expression. To test this model, we infected two different MAGI cell lines with virus particles pseudotyped with the X4-tropic NL4-3 envelope. While both MAGI cell lines express CXCR4 and CD4, only the MAGI-R5 cells are engineered to co-express CCR5. Infection with X4-tropic virus particles was inhibited by BFFI only on the MAGI-R5 cells. (Figure 2E). In contrast, the fusion inhibitor T-2635 blocked infection of the X4-tropic virus particles efficiently in both cells lines, independent of the CCR5 co-expression (data not shown). These data support a model in which BFFI can only exert the antiviral effect against X4 viruses on cells co-expressing CCR5, such as JC53BL or MAGI-R5 cells, but not in cells with no CCR5 expression such as MAGI-X4. Based on these observations, we suggest that anchoring of the CCR5mAb to the CCR5 receptor is a pre-requisite for BFFI activity against X4 viruses. In this model, anchoring of BFFI increases the local concentration of the fusion inhibitor on the cell surface in close proximity to its target, the viral envelope protein gp41.

To confirm the anchoring hypothesis, we pre-incubated JC53BL cells (expressing all surface receptors CD4, CCR5 and CXCR4 at high levels) with saturating concentrations of either the CCR5 antagonist maraviroc (2 μM) or the CCR5mAb (3 μg/ml). Following the 45 min pre-incubation, cells were infected with X4-tropic virus particles in the presence of BFFI. Maraviroc and CCR5mAb both bind to the CCR5 receptor but do not prevent infection by X4-tropic virus particles. Pre-incubation with maraviroc does not significantly alter the susceptibility of BFFI against the X4-tropic virus particles (Figure 2F). We have shown previously that the binding of small molecule antagonists to the transmembrane domains of CCR5 does not interfere with the binding of a CCR5mAb, targeted to the extracellular loops of the receptor [14,16]. In contrast, pre-binding of the CCR5mAb which competes with BFFI for binding to CCR5 receptors, resulted in a 90-fold increase in the IC_50 _for BFFI compared to pre-incubation with medium only (Figure 2F).

To confirm the anchoring hypotheses in the biologically relevant human PBMC, we infected PBMC with replication competent, X4 tropic virus NL4-3 in presence of BFFI or T-2635 and analyzed the viral cytopathic effect on the CCR5(+) and CCR5(-) subset of CD4(+) T-cells. The cytopathic effect was measured as the depletion of CD4(+) T-cells relative to CD8(+) T-cells, which are not infected or lysed by HIV-1 [25]. Infection with NL4-3 reduced the CD4/CD8 ratio in both the CCR5(+) and CCR5(-) subset of CD4(+) T-cells by approximately 75–80% compared to uninfected control cells. T-2635 protected CD4(+) T-cells from NL4-3-induced depletion, independent of the CCR5 expression. In contrast, BFFI prevented CD4 depletion only in the subset of CCR5-expressing, but not in the CCR5(-) cells (Figure. 2G). Since the majority of CD4(+) T-cells are CCR5(-), the protective effect of BFFI was undetectable in the total un-gated CD4(+) T-cells. We conclude from this experiment that BFFI would be protective only on the small subset of CCR5-expressing PBMC, where anchoring of the CCR5mAb allows BFFI to be in close proximity to the X4 viral fusion and prevents virus from infection and subsequent depletion of CD4 (+) T-cells.

## Discussion

Here we describe the concept of an antiretroviral agent with dual mechanisms of action. The bifunctional fusion inhibitor, BFFI, is an antibody-based entry inhibitor that combines the activity of an anti-CCR5 monoclonal antibody with the activity of a fusion inhibitor in one molecule. It was constructed by expression of a genetic construct coding for the fusion peptide, T-2635, a short peptide linker and an anti-CCR5 antibody RO-mA0630, a mouse/human chimera of the previously described CCR5mAb ROAB14 [14]. The BFFI entity showed potent antiviral activity in the single cycle entry assays, as well as in the biologically relevant PBMC assay for the R5 tropic viruses tested. In a series of experiments we demonstrated that both the CCR5mAb and the fusion inhibitor of BFFI contribute to the antiviral activity. BFFI has an increased antiviral activity compared to IGF-R1mAb-FI, where the two molecules differ only in the variable regions of the antibody, one recognizing IGF-1R and the other the HIV co-receptor CCR5 (Table 1). Several lines of evidence were generated to support the contribution of the fusion peptide to the antiviral potency of BFFI. We demonstrated that the antiviral potency of BFFI against the X4-tropic viral isolate 098-FI^res ^is reduced to the same extend as the susceptibility to T-2635. Analysis of the contribution of the CCR5 mAb and FI components within the sequential steps of viral fusion in synchronized viral infection experiments further demonstrated activity of the fusion peptides, as BFFI showed viral inhibition closer to the fusion peptide component alone with measurable times of 40 and 48 minutes respectively in comparison to the CCR5 mAb alone, which was measured at 18 minutes.

**Figure 3:**
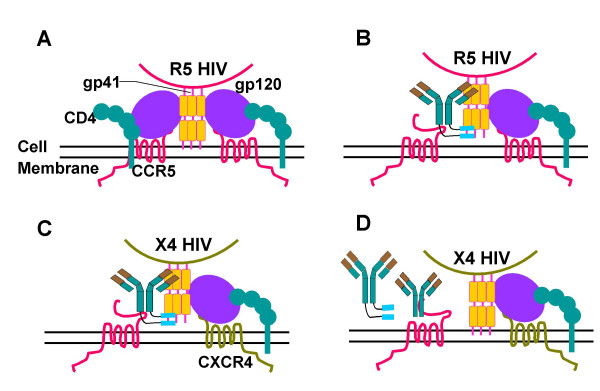
Proposed model of the mechanism of action of BFFI. The fusion process of an R5 virus with the host cell is depicted in Figure **A**. HIV entry begins with an initial contact of between the viral envelope protein gp120 and the cell surface receptor CD4, followed by gp120 binding to the co-receptor CCR5. This leads to conformational changes in gp120 and the insertion of HIV gp41 N-terminal fusion peptides into the cell membrane. The C-helical heptad repeats (C-HR) and N-helical heptad repeats (N-HR) form a six helix bundle bringing viral envelope and cell membrane in close proximity and resulting in fusion. **B**. During an R5 HIV infection in the presence of BFFI, binding of BFFI to CCR5 markedly enhances the cell surface concentration of FI and possibility of interference with HIV gp41. **C**. During an X4 HIV infection in cells that express both CCR5 and CXCR4 in the presence of BFFI, the same result is obtained. If the X4 virus is able to enter the fusion stage by binding to nearby CD4 and CXCR4, the FIs within BFFI can effectively inhibit the six-helix bundle formation. **D**. If cells are pre-coated with CCR5mAb, BFFI is no longer able to bind to CCR5 and will not be effective at blocking the X4 HIV fusion.

While BFFI is active against X4-tropic viruses in recombinant cells expressing high levels of CCR5, infection of PBMC with X4-tropic viruses could not be prevented. We explored the mechanism of action for BFFI in competition and cell population experiments. In the absence of CCR5 expression on the target cell, the high molecular weight of BFFI results in decreased diffusion and access of the FI portion to the six-helix bundle formation during the fusion process (Figure 3a). Binding of BFFI to the CCR5 receptor however greatly increases the local concentration of available fusion peptide in close proximity to the viral entry process on the cell surface and its ability to block the viral fusion process (Figure 3b). This model for viral inhibition is supported in competition experiments where pre-incubation with CCR5mAb resulted in a substantial drop in the ability of BFFI to block X4-tropic viral cell entry (Figure 3d).

Lack of antiviral efficacy against replication competent X4 or dual tropic virus in PBMC can be explained by the distribution of CCR5 and CXCR4 receptors on various sub-populations of human PBMC. Expression of CCR5 on the CD4(+) T-cell population of PBMC is limited to a small subset of cells, whereas CXCR4 is expressed on most of the CD4(+) T-cells [23,24]. Therefore, BFFI can inhibit depletion by X4-tropic viruses only on the small subset of CCR5- and CXCR4-expressing CD4(+) T-cells, but is ineffective on the majority of CCR5-negative CD4(+) T-cells. As an extension of our approach to generate a bifunctional fusion inhibitor entity, block of all HIV-1 viruses in a tropism-independent manner should be the goal in the next generation BFFIs.

HAART (highly active antiretroviral therapy) is the current standard of care for HIV-infected patients. Common challenges with HAART are pill burden, durability, adverse drug-drug interactions, and toxicities. The concept of a bifunctional HIV entry inhibitor, as described here, is the prototype of an antiretroviral agent that has the potential to address several of these limitations. Could the increased potency, observed with the synergistic activity of two pharmacophores in one molecule, lead to prolonged durability? In addition, the incorporation of two inhibitors into one molecule with exquisite potency and the naturally occurring antibody framework may have potential for low and infrequent dosing regimens and fewer drug-drug interactions. In viral selection experiments, viruses resistant to 2^nd ^generation fusion inhibitors such as T-2635 could not yet be isolated *in vitro *[18]. Similarly, a viral isolate resistant to the CCR5 monoclonal antibody Pro-140 could only be isolated after prolonged *in-vitro *passaging and showed only modest loss in susceptibility to Pro-140 [26]. Combining the activity of T-2635 and a CCR5mAb in one molecule could further increase the genetic hurdle for developing resistance to the combined agent. Viruses resistant to the fusion inhibitor part of the BFFI may still be susceptible to the CCR5mAb activity, and *vice versa*. Co-evolution of both resistance pathways may have fitness implications for the viral envelope and replication cycle.

In conclusion, BFFI is a prototype for a novel type of antiretroviral agents with a dual mode of action. Expanding this concept to X4- and dual-tropic viruses while maintaining the antiviral potency will be explored further.

## Materials and methods

### Reagents, viruses and cell lines

JC53BL (TZM-bl) cells were obtained from the NIH AIDS Research and Reference Reagent program. Human embryonic kidney cells 293-T and HEK293-EBNA cells were obtained from ATCC, Manassas, VA. Human PBMC were obtained from AllCells (Emeryville, CA), stimulated for one day in PBMC media (RPMI-1640 media containing 10% fetal bovine serum (FBS), 1% penicillin/streptomycin, 2 mM L-glutamine, 1 mM sodium-pyruvate, 0.1 mM MEM non-essential amino acids) supplemented with 2 μg/mL phytohemagglutinin (all Invitrogen) and maintained in PBMC media containing 5 units/mL human IL-2 (Roche Applied Science, Indianapolis, IN).

NL4-3 and 92US715 were obtained from the NIH AIDS Research and Reference Reagent program. YU2.c was obtained from Dr. G. Shaw, University of Birmingham, Alabama. JRCSF was obtained from Dr I. Chen, University of California, Los Angeles. NL-Bal was obtained from Roche Welwyn, UK. Viruses 89.6 was purchased from ABI, Colombia, MD. CC1/85 was a gift from Dr. C. Stoddart (The J. David Gladstone Institutes, San Francisco, CA). W969-2, W969-5 and W969-7 were a gift from Dr. D. Richman, University of California, San Diego. Viruses 098 and 098-FI^res ^(previously known as 098–1144) were gifts from Dr. M. Greenberg (Trimeris, Morrisville, NC).

The N-terminally acetylated and C-terminally amidated T-2635 peptide was a gift from Dr. M. Greenberg (Trimeris, Morrisville, NC). It was synthesized as described in [18].

### Expression plasmids

Antibody light and heavy chain genes were expressed from 2 separate plasmids. Expression of all antibody and antibody-FI light and heavy chains is controlled by a shortened intron A-deleted immediate early enhancer and promoter from the human cytomegalovirus (HCMV). Coding sequences are followed by the native human immunoglobulin κ-polyadenylation signal sequence (light chain) or the native human γ1-immunoglobulin polyadenylation signal sequence (heavy chain). The structural gene of the IGF-1RmAb light chain is assembled by fusing the cloned human IGF-1RmAb variable light chain cDNA including the native light chain signal sequence with the human κ-light gene constant region. The structural gene of the human IGF-1RmAb heavy chain is assembled by fusing the cloned human IGF-1RmAb variable heavy chain cDNA with a DNA segment coding for a murine immunoglobulin heavy chain signal sequence including a signal sequence intron (L1, intron, L2) and at the 3'-end with a DNA segment containing a splice donor site and a unique NotI restriction site. The NotI restriction site enables the joining to the genomic human γ1-heavy gene constant region including the mouse Ig μ-enhancer and a slightly modified CH_3_-IgG_1 _polyadenylation (pA) joining region: A unique HindIII and NheI restriction site was created to allow the insertion of peptide linker and peptide fusion inhibitor encoding DNA-fragments in frame to the heavy chain. The peptide linker sequence and the N-terminally extended T-2635 peptide sequence (NN-) are shown in Fig. 1A. The structural gene of the chimeric mouse/human CCR5mAb light and heavy chains are assembled in similar ways.

### Transient expression and purification of proteins

IGF-IRmAb, IGF-IRmAb-FI, CCR5mAb and CCR5mAb-FI were expressed by transient transfection of HEK293-EBNA cells. The CCR5mAb was also expressed by a stably transfected mouse myeloma cell line NSO. Cells were co-transfected with plasmids encoding the light chain and heavy chain, respectively at ratios from 1:2 to 2:1. MAb and mAb-FI-containing cell culture supernatants were harvested at day 4 to 11 after transfection.

MAbs and mAb-FIs were captured by affinity chromatography from clarified culture supernatants using Protein A-Sepharose™ CL-4B (GE Healthcare) equilibrated with PBS buffer. Unbound proteins were washed out with PBS equilibration buffer and 0.1 M citrate buffer, pH 5.5. Then, the mAbs and mAb-FIs were eluted with 0.1 M citrate buffer, pH 3.0, and the protein containing fractions were immediately neutralized with 1 M Tris-Base. Size exclusion chromatography (SEC) on Superdex 200™ (GE Healthcare) was used as a second purification step. The eluted mAbs and mAb-FIs were concentrated and stored at -80°C.

### Analytic characterization of proteins

The protein concentration of mAb and mAb-FI samples was determined by measuring the optical density (OD) at 280 nm, using the molar extinction coefficient calculated on the basis of the amino acid sequence. The purity and the proper tetramer formation of mAbs and mAb-FIs were analyzed by SDS-PAGE in the presence and absence of a reducing agent (5 mM 1,4-dithiotreitol) and staining with Coomassie brilliant blue. The aggregate content of mAb and mAb-FI samples was analyzed by high-performance SEC using a TSK3000SWxl analytical size-exclusion column (TosoHaas, Stuttgart, Germany). The integrity of the amino acid backbone of reduced mAb and mAb-FI light and heavy chains were verified by NanoElectrospray Q-TOF mass spectrometry after removal of N-glycans by enzymatic treatment with Peptide-N-Glycosidase F (Roche Molecular Biochemicals).

### Stable Expression of human CCR5 in U373-MAGI-CXCR4CEM Cells

FuGene6 (Roche Applied Science) transfection reagent was used to transfect pCDNA3.1_hCCR5 [14] into U373-MAGI-CXCR4CEM cells (Cat.# 3956, NIH AIDS Research & Reference Reagent Program, Germantown, MD 20874) following manufacturer's instructions. Forty-eight hours after transfection, Zeocin (Invitrogen, Carlsbad, CA) was introduced into the medium to the final concentration of 400 μg/mL to select for CCR5-expressing cells. Cells expressing CCR5 at high and low expression level were enriched by several rounds of FACS sorting.

#### Single cycle entry assay

To generate pseudotyped viral particles for infection of the reporter cell line JC53BL, 293T cells were co-transfected with pNL4-3Δenv (pNL4-3 with a deletion of the envelope gene) and the expression vector pCDNA3.1 (Invitrogen) encoding either the envelope gene of NLBal or NL4-3. To generate virus particles for the infection of MAGI cells, 293T cells were co-transfected with pNL4-3Δenv-luc (pNL4-3Δenv with a luciferase gene cloned into the nef open reading frame) and the envelope-expression plasmid. Cell culture supernatants containing pseudotyped viral particles were harvested, filtered, stored at -80°C and titered on JC53BL or MAGI cells, respectively. To test for antiviral potency, compounds were diluted in quadruplicates in white 96-well-plates (Greiner Bio-one, Frickenhausen, Germany). The equivalent of 1 × 10^5 ^relative light units of virus particles were used to infect 25.000 JC53BL or MAGI cells/well in a total volume of 200 μl. After incubation for 3 days, 50 μl Steady-Glo^® ^luciferase reagent (Promega, Madison, WI) were added, incubated for 5 min and the plates were read using a Luminoskan (Thermo Electron Corporation, Waltham. MA). The IC_50 _was determined using the sigmoidal dose-response model with one binding site in Microsoft XLfit.

#### Antiviral Assay

Antiviral potencies for Tab. 2 and Fig. 2c were generated by infecting 25.000 JC53BL cells with replication-competent virus as described for the single cycle entry assay. Viruses were amplified using PBMC. Viral replication was monitored by infecting JC53BL cells and subsequent luciferase read-out. Cell culture supernatants containing pseudotyped viral particles were harvested, filtered and stored at -80°C. To test for antiviral potency, compounds were diluted in quadruplicates in white 96-well-plates (Greiner Bio-one, Frickenhausen, Germany). The equivalent of 1 × 10^5 ^relative light units of virus particles were used to infect 25.000 JC53BL cells/well in a total volume of 200 μl. After incubation for 3 days, 50 μl Steady-Glo^® ^luciferase reagent (Promega, Madison, WI) were added, incubated for 5 min and the plates were read using a Luminoskan (Thermo Electron Corporation, Waltham. MA). The IC_50 _was determined using the sigmoidal dose-response model with one binding site in Microsoft XLfit.

#### Time of addition experiments

For the time course infection study, MAGI-R5 cells (6 × 10^4^/well) were seeded in 24-well plates overnight. HIV-1 pseudotyped viruses were chilled at 4°C for 20 min and added into pre-chilled MAGI-R5 cells. Spinoculation was performed by spinning at 2000 rpm at 4°C for 1 h. The cells were washed once with cold PBS and then followed by 450 μl of medium at 37°C. At various time points, CCR5 inhibitors at IC_90 _– IC_95 _concentrations were added to the cells, in 50 μl of medium containing 0.5% FBS. Luciferase activity was measured 48 h post-infection and % virus entry for each time point was calculated as (RLU with inhibitor)/(RLU without inhibitor) × 100.

#### PBMC assay

Human PBMC were isolated from buffy-coats (obtained from the Stanford Blood Center) by a Ficoll-Paque centrifugation. PBMC were treated with 2_μg/ml Phytohemagglutinin (Invitrogen, Carlsbad, CA) for 24 h at 37°C, then with 5 Units/ml human IL-2 (Roche Applied Sciences, Indianapolis, IN) for a minimum of 48 h prior to the assay. In a 96 well round bottom plate, 1 × 10^5 ^PBMC were infected with 800 pg p24 of the indicated HIV-1 strain in the presence of serially diluted inhibitor. Plates were incubated for 6 days at 37°C. Virus production was measured at the end of infection by using p24ELISA (PerkinElmer) according to the manufacturer's instruction. IC_50 _was determined using the sigmoidal dose-response model with one binding site in Microsoft XLfit.

### Determination of receptor surface expression by flow cytometry

The CD4, CCR5 and CXCR4 receptor expressions were assessed on MAGI-huCCR5-high, MAGI-huCCR5-low, JC53-BL cell lines and normal human PBMC. Antibodies bound per cell (ABC) were analyzed with flow cytometry using Becton Dickinson QuantiBRITE PE beads (Cat# 340495, BD, San Jose, CA) following manufacturer's instructions. Antibody-PE conjugates (1:1) specific to CD4, CCR5 and CXCR4 were custom ordered from BD Pharmingen (BD, La Jolla, CA). Molecules per particle were calculated using the QuantiBRITE beads as standards according to manufactures instructions.

### CD4+ cell depletion

In round-bottom 96 well plates, 1.5 × 10^5 ^PBMC were infected with 50 TCID_50 _units of the NL4-3 virus in presence of 5 ug/mL BFFI or 5 ug/mL T-2635 and incubated for five days at 37°C. At day 5 post-infection, cells from three wells of each condition were washed once with 200 μl FACS-buffer (phosphate-buffered saline (Invitrogen) supplemented with 2% FBS) and stained with the anti-CD3 monoclonal antibody SK7, fluorescein isothiocyanate (FITC) conjugated and with the anti-CD8 monoclonal antibody SK1, peridimin chlorophyll protein (PerCP) conjugated for 30 minutes are room temperature. To stain for CCR5 pre-bound by BFFI, the CCR5mAb ROAb13 [14] that recognizes the N-terminus of the CCR5 was used. ROAb13 was allophycocyanin (APC) labeled using the Alexa Zenon labeling kit (Invitrogen-Molecular Probes). After antibody staining, cells were washed twice with PBS, fixed in 1% paraformaldehyde and subjected to flow cytometry analysis using a BD FACScalibur (BD Biosciences, San Jose, CA). A total of 100,000 lymphocytes positive for CD3 surface marker were acquired per sample and the data were analyzed with Cellquest software (Becton Dickinson). CD4 T-cell depletion was assessed by measuring the ratio of CD4 to CD8 T cells. This value was normalized to the CD4/CD8 ratio of control (uninfected) samples.

## Abbreviations

BFFI: bifunctional entry inhibitor; HIV-1: human immunodeficiency virus type 1; HAART: highly active antiretroviral therapy; FI: fusion inhibitor
